# Safety of magnetic resonance imaging in patients with cardiac implantable electronic devices with generator and lead(s) brand mismatch

**DOI:** 10.1002/acm2.13520

**Published:** 2022-01-23

**Authors:** Nareg Minaskeian, Sofia P Hajnal, Michael B Liu, Lindsay M Klooster, Katrina L Devick, Linda Schwartz, Clinton E Jokerst, Dan Sorajja, Luis RP Scott

**Affiliations:** ^1^ Department of Electrophysiology Mayo Clinic Arizona Phoenix Arizona USA

**Keywords:** cardiac implantable electronic device, MRI, safety

## Abstract

Magnetic resonance imaging (MRI) is a valuable imaging modality for the assessment of both cardiac and non‐cardiac structures. With a growing population of patients with cardiovascular implantable electronic devices (CIEDs), 50%–75% of these patients will need an MRI. MRI‐conditional CIEDs have demonstrated safety of MRI scanning with such devices, yet non‐conditional devices such as hybrid CIEDs which have generator and lead brand mismatch may pose a safety risk. In this retrospective study, we examined the outcomes of patients with hybrid CIEDs undergoing MRI compared to those patients with non‐hybrid CIEDs. A total of 349 patients were included, of which 24 patients (7%) had hybrid CIEDs. The primary endpoint was the safety of MRI for patients with hybrid CIEDs as compared to those with non‐hybrid devices, measured by the rate of adverse events, including death, lead or generator failure needing immediate replacement, loss of capture, new onset arrhythmia, or power‐on reset. Secondary endpoints consisted of pre‐ and post‐MRI changes of decreased P‐wave or R‐wave sensing by ≥50%, changes in pacing lead impedance by ≥50 ohms, increase in pacing thresholds by ≥ 0.5 V at 0.4 ms, and decreasing battery voltage of ≥ 0.04 V. The primary endpoint of any adverse reaction was present in 1 (4.2%) patient with a hybrid device, and consistent of atrial tachyarrhythmia, and in 10 (3.1%) patients with a non‐hybrid device, and consisted of self‐limited atrial and non‐sustained ventricular arrhythmias; this was not statistically significant. No significant differences were found in the secondary endpoints. This study demonstrates that MRI in patients with hybrid CIEDs does not result in increased patient risk or significant device changes when compared to those patients who underwent MRI with non‐hybrid CIEDs.

## 1 INTRODUCTION

**FIGURE 1:**
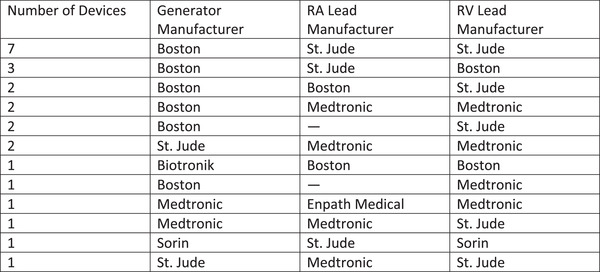
A summary of the generator, RA lead, and RV lead manufacturers for the 24 hybrid devices. Squares with “—” are those without an RA lead. No hybrid devices included LV leads

Magnetic resonance imaging (MRI) is a valuable imaging modality for the assessment of both cardiac and non‐cardiac structures for a variety of pathological processes; it provides improved diagnostic yield compared to computed tomography as well as the advantage of not exposing the patient to ionizing radiation.^1^, ^2^ Furthermore, with a growing population of patients with cardiovascular implantable electronic devices (CIEDs), by as much as 55.6% between 1993 and 2009, it is estimated that about 50%–75% of these patients will need an MRI during their lifetime.^1^, ^3^ In addition, MRI is currently considered the gold standard imaging exam for a series of cardiologic diseases, such as myocardial viability after infarction or myocardial function,^4^ which makes it even more important that patients with such devices may undergo the examination. Figure 1

Until the advent of MRI‐conditional CIEDs in 2008, MRI was traditionally considered a contraindication in patients with CIEDs. The use of a static magnetic field, gradient magnetic field, and radiofrequency pulses can heat and possibly damage the various components within a CIED, resulting in harm. Possible effects from exposure to the fields include heating or movement of the CIEDs, electrical reset, device malfunction, arrhythmias, and even death.^5^, ^6^ However, a growing preponderance of evidence has shown that MRI in patients with non‐MRI conditional CIEDs is feasible. The 2017 Heart Rhythm Society expert consensus statement provided a Class IIa recommendation (level of evidence B‐NR) for MRI of non‐conditional CIEDs^7^; however, this recommendation takes into account that certain conditions are met and protocols are followed.

As defined by the United States Food and Drug Administration (USFDA), an MRI conditional device has demonstrated safety in the MR environment within defined conditions, and at a minimum, addresses the conditions of the static magnetic field, the switched gradient magnetic field, and the radiofrequency fields. These requirements may be found on the USFDA guidance document docket FDA‐2019‐D‐2837.

MRI scanners create and expose the patient to powerful magnetic fields including static magnetic fields, radiofrequency fields, and gradient magnetic fields.^6^ The static magnetic field effect may lead to reed switch activation reverting the device to an asynchronous pacing mode, may reset the device, and has the potential to move the device or dislodge leads.^6^ Studies have shown that the force created by a 1.5 T MRI machine to be 0.05–3.6 N for pacemakers, and 1–5.9 N in internal cardioverter–defibrillators.^1^

The radiofrequency field is generated after the static magnetic field has been created, and this may lead to heating of the generator and lead causing thermal tissue injury, as well as lead malfunction manifested by increased pacing thresholds.^6^ It has been demonstrated that the temperature rise is on a spectrum, from little to no rise up to a rise by 20°C in vivo, with three reports finding no evidence of tissue injury.^1^ Although we were unable to perform temperature measurements, there was no clinical indication of such effect.

Throughout MRI scanning, the machine creates a gradient field, and this has the potential to induce currents within the leads mimicking intrinsic activity causing inhibition of pacing, high pacing rates, inappropriate administration of therapies, and the induction of possibly fatal arrhythmias such as ventricular tachycardia or fibrillation.^6^

This retrospective study was done to assess the safety of undergoing MRI in patients with hybrid CIEDs, compared with those with non‐hybrid CIEDs, and found there are no significant changes in outcomes associated with patient safety or device and lead function.

## 2 METHODS

### 2.1 Methods

We conducted a retrospective study using a database consisting of any patient with a CIED who had undergone any clinically indicated MRI at our institution from March 2014 through December 2019. Patients who did not have data on the pre‐ and post‐MRI device interrogation were excluded. The study was approved by our Institutional Review Board. These patients had undergone same day device interrogation pre‐ and post‐MRI through which the device type was ascertained, and generator and lead function was evaluated.

Our patient cohort consisted of those who had a hybrid CIED with generator and lead brand mismatch; and the control group consisted of those with brand consistent non‐hybrid CIEDs, irrespective of MRI‐conditional status. A total of 427 pacemakers, 130 defibrillators, and 3 abandoned leads were included in the study; however, no fractured or extracardiac epicardial leads were included. Transvenous epicardial leads (such as those placed in the coronary sinus for cardiac resynchronization therapy) were included. MRI was performed for locations including the brain, head and neck, chest, abdomen and pelvis, upper and lower limbs, and the spine; however, due to most MRIs having such few hybrid device observations, we considered all MRIs together and did not run separate analyses for the different types of MRI.

Our institutional safety protocol required same day device interrogations pre‐ and post‐MRI, with patient observation during the MRI by the device nurse specialist, and an electrophysiologist on‐call at the hospital. In patients with MRI‐conditional devices, the “MRI Mode” was selected (which also turns off therapy delivery in defibrillators), and in pacer‐dependent patients an asynchronous pacing mode was selected at a rate higher than the intrinsic sinus (such as ventricular, off, off (VOO)/dual, off, off (DOO)), and in non‐dependent patients a back‐up pacing mode was selected at 40 bpm (such as DDI/VVI). In patients with non‐conditional devices, therapies were turned off for defibrillators, and the same pacing modes were selected for pacing‐dependent and non‐dependent patients. A standard 1.5‐T clinical MRI machine was used for scanning. Endpoints were explicated by Russo.^8^ The primary endpoint was the safety of MRI for patients with hybrid CIEDs as compared to those with non‐hybrid devices, measured by the rate of adverse events, including death, lead or generator failure needing immediate replacement, loss of capture, new onset arrhythmia, or power‐on reset. The secondary endpoints consisted of pre‐ and post‐MRI changes of decreased P‐wave or R‐wave sensing by ≥50%, changes in pacing lead impedance by ≥50 ohms, increase in pacing thresholds by ≥ 0.5 V at 0.4 ms, and decreasing battery voltage of ≥ 0.04 V.

### 2.2 Statistical analysis

The data were summarized using descriptive statistics comparing hybrid and non‐hybrid devices. Continuous variables were compared using the Wilcoxon rank sum test and categorical variables were compared using the Fisher's exact test for count data. Since the majority of the information recorded was on the first MRI since implantation and since multiple MRIs on the same device are likely dependent, we focused our analyses only on the first MRI since implantation. The primary outcome we considered were adverse events, and differences in lead impedance and threshold, P‐ and R‐wave sensing, and battery voltage pre‐/post‐MRI were considered as secondary outcomes. For all pre‐ and post‐MRI measures, we defined the change as the post‐MRI measurement minus the pre‐MRI measurement, so any negative difference is an observed decrease, and any positive difference is an observed increase in the variable after undergoing MRI.

We compared the distribution of each outcome variable for hybrid and non‐hybrid devices to determine if we had evidence that hybrid devices had similar reactions to non‐hybrid devices when undergoing MRI. To do this, for each outcome variable, we estimated the location difference of the distribution for hybrid and non‐hybrid devices and the corresponding 95% confidence interval for this difference. Since most of the distributions of the outcome measurements were skewed and there were limited hybrid observations, we used the Wilcoxon rank sum test and corresponding confidence interval to compare the distribution of hybrid and non‐hybrid devices. This is a nonparametric test similar to the two independent sample *t*‐test. For all tests, *P* < 0.05 was considered statistically significant.

## 3 RESULTS

A total of 349 patients with CIEDs underwent a first MRI scan since implantation, of which 24 patients (7%) had hybrid CIEDs implanted (Figure 1) and 325 (93%) did not. The primary endpoint of any adverse reaction was present in 1 (4.2%) patient with a hybrid device, and consistent of self‐limited atrial tachyarrhythmia, and in 10 (3.1%) patients with a non‐hybrid device, and consisted of self‐limited atrial and non‐sustained ventricular arrhythmias (OR: 1.37, 95% CI 0.03–10.41, *P* = 0.55). No deaths, lead or generator failure, loss of capture, or power‐on reset occurred.

With respect to the secondary endpoints, the median percent change of atrial lead impedance was −1.57 in the hybrid arm compared to 0.00 in the non‐hybrid arm (*P* = 0.12). The median percent change in right ventricular lead impedance was −1.66 in the hybrid arm, and 0.00 in the non‐hybrid arm (*P* = 0.55). The median percent change in left ventricular lead impedance was 3.19 in the hybrid arm, and 0.00 in the non‐hybrid arm (*P* = 0.39). The median percent change of P‐wave sensing was −8.33 in the hybrid arm compared to 0.00 in the non‐hybrid arm (*P* = 0.33). The median percent change of R‐wave sensing was 0.00 in both the hybrid arm and non‐hybrid arm (*P* = 0. 42). The median percent change of atrial lead pacing threshold was 0.00 in both the hybrid and non‐hybrid arms (*P* = 0.83), the median percent change of right ventricular lead pacing threshold was 0.00 in both the hybrid and non‐hybrid arms (*P* = 0.54), and the median percent change of left ventricular lead pacing threshold was 14.29 in the hybrid arm and 0.00 in the non‐hybrid arm (*P* = 0.11). The median percent change of the battery voltage was 0.000 in both arms (*P* = 0.01); while the medians of 0.000 are the same, the there is evidence that the distributions are different. Of note, three patients with non‐hybrid CIEDs had an abandoned right atrial or right ventricular lead, and none of those patients experienced any primary adverse events.

## 4 DISCUSSION

In our study, we evaluated the safety of patients with implanted hybrid CIEDs who underwent clinically indicated MRI.

MRI creates powerful magnetic fields including static magnetic fields, radiofrequency fields, and gradient magnetic fields, which may pose danger to patients who have CIEDs.^6^ Pavlicek in 1983 was the first to discuss the effects of these fields on pacemaker devices using ex vivo pacemakers in deceased patients.^9^ Since then, there have been multiple studies elucidating the effects of MRI on CIEDs. The main risks include induced electrical currents, arrhythmia induction, thermal heating of leads, and device malfunction including changes of impedance, sensing, pacing thresholds, and battery voltage.^10^

Since then, multiple changes in the device and leads design have been made to reduce risks of adverse events in patients with CIEDs undergoing MRI, such as: ferromagnetic content was minimized; reed switches were modified (in older models); leads were redesigned to reduce induced currents/heating; circuitry filters and shielding were implemented to impede or limit the transfer of certain unwanted electromagnetic effects.^11^ These changes have contributed to making MRI safer for patients with CIEDs.

The current studies have compared MRI‐conditional CIEDs and non‐conditional CIEDs, with the same primary and secondary endpoints as this study, and have demonstrated that performing MRI scanning on patients with non‐MRI conditional CIEDs is feasible with little risk; giving rise to the Class IIa recommendation by the Heart Rhythm Society for MRI in patients with non‐conditional CIEDs.^2^, ^7^, ^12^, ^13^ While some of these studies did include hybrid CIEDs, to the best of our knowledge, there are no studies which compare the safety of MRI on hybrid CIEDs to non‐hybrid CIEDs.

Our study revealed that MRI in those patients with hybrid CIEDs is feasible, and there is no difference in safety or device function when compared to those patients who underwent MRI with non‐hybrid CIEDs. There were no deaths, lead or generator failure, loss of capture, or power‐on reset; and furthermore, with respect to the secondary endpoints, no significant changes in lead impedance, P‐ or R‐wave sensing, pacing thresholds, or battery voltage occurred.

## 5 LIMITATIONS

The limitations of our study should be noted. First, our study was retrospective and the number of patients with hybrid CIEDs was less than the number of patients with non‐hybrid CIEDs. Furthermore, given that the data were extracted from a database, some patients did not have the complete data we are seeing. Second, our patient cohorts did not include those with fractured or extracardiac epicardial leads, thus these results may not be extrapolated to include these patients. Moreover, data for high‐voltage lead impedance was not investigated. Third, the MRIs were performed using a 1.5 T MRI machine, and the results may not be extrapolated to those machines using a higher field strength. Finally, defibrillation threshold testing was not performed post‐MRI, which due to our findings of no significant changes in device and lead function, we found admissible.

## 6 CONCLUSION

The results found in this study are consistent with other studies that compared MRI‐conditional CIEDs with non‐conditional CIEDs,^14^ however the other studies did not compare the safety of hybrid CIEDs to non‐hybrid CIEDs. This study demonstrates that MRI in patients with hybrid CIEDs does not result in increased patient risk or significant device changes when compared to those patients who underwent MRI with non‐hybrid CIEDs. Nevertheless, it is important for physicians to understand the complications that may be associated with MRI scanning in any patient with a CIED, regardless of the type of CIED implanted.

## CONFLICTS OF INTEREST

The authors declare that there is no conflict of interest that could be perceived as prejudicing the impartiality of the research reported.

## AUTHOR CONTRIBUTIONS

Nareg Minaskeian: Data collection, writing and revision of manuscript, reference review, and statistical analysis. Sofia Pannunzio Hajnal: Data collection, writing and revision of manuscript, and reference review. Lindsay Klooster, Michael Liu and Linda Schwartz: Data collection. Katrina Devick: Statistical analysis and writing of manuscript. Clinton E Jokerst: Revision of manuscript. Dan Sorajja: Revision of manuscript. Luis RP Scott: Principal investigator, revision of manuscript.

## Data Availability

The data that support the findings of this study are available from the corresponding author upon reasonable request.
